# medAR: An augmented reality application to improve participation in health‐care decisions by family‐based intervention

**DOI:** 10.1111/hex.12981

**Published:** 2019-09-30

**Authors:** Yan Li, Jinzhi Li, Jing Zhang, Guoliu Ye, Zhengmei Zhou

**Affiliations:** ^1^ Department of Pathophysiology Bengbu Medical College Bengbu China; ^2^ Department of Nursing Bengbu Medical College Bengbu China; ^3^ Department of Gynecology The First Affiliated Hospital of Bengbu Medical College, and Obstetrics Bengbu China; ^4^ Teacher Development Center Bengbu Medical College Bengbu China

Dear Editor,

I have read the review article entitled ‘Interventions to improve participation in health‐care decisions in non‐Western countries: A systematic review and narrative synthesis’ by Hankiz Dolan et al^1^ I want to congratulate the authors for this thoughtful article and for making some contributions.

As a medical educator in China, I think I should add personal comments to the discussion. Firstly, I would like to revise his statement slightly, to read: ‘We acknowledge that family and significant others can play a significant role in the process of decision making in some patients from non‐Western *or Western* cultural backgrounds *more or less*.’^2^ [my emphasis].

Secondly, we equally know that doctors do not fulfil the obligation of medical information provision from the perspective of Chinese patients.^3^ We manage to change information asymmetry between doctor and patient or family member. Several studies have shown that augmented reality (AR) can assist shared decision making (SDM)^4^, ^5^: AR can support doctors’ explanations with a live simulation that presents all the involved clinical processes.

So, we created AR content using the RAVVAR (https://ravvar.us/) model, an easy‐to‐learn non‐programming editor tool for AR. We used RAVVAR in PC combined health‐care needs to create AR content for topics related to health‐care education (eg integral nursing for a cesarean section: an eNurse named ‘Xiao Jing’ can provide health‐care education on patient's physical and mental health before, during and after the operation). We then exported the content into the medAR application (Figure 1). The users of medAR can experience a combination of virtual scenarios and real situations; there are multiple interactive steps and quizzes; finally, there is a link to the online examination.

**Figure 1:**
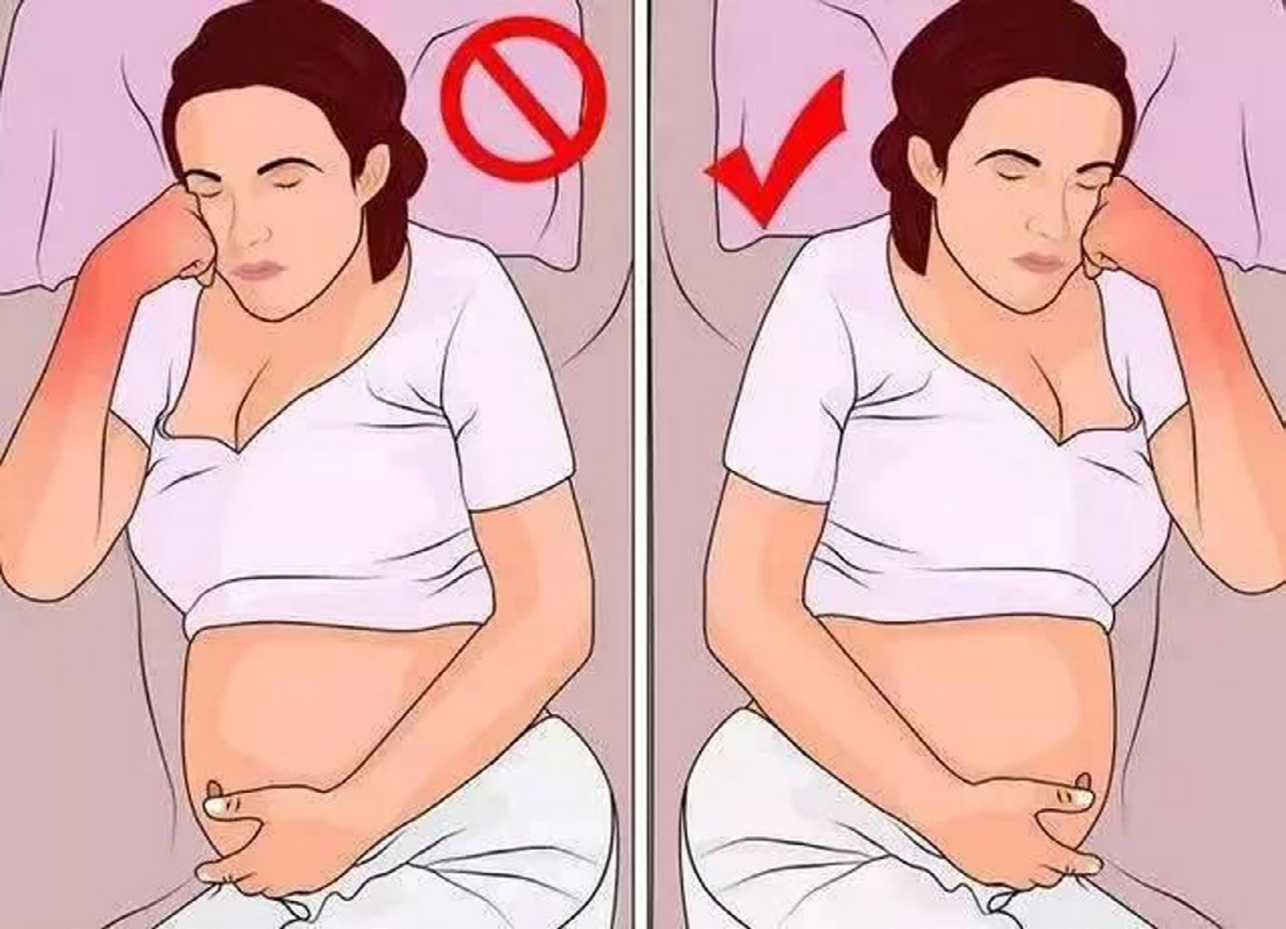
Download medAR and open it to scan the marker image to experience the AR content. The medAR is available to download from https://ravvar.cn/app/medar. If a couple of users open the phone app medAR at the same time, one can experience AR through scanning the marker in another phone

Then in family caregivers of cesarean section maternity, six participators (control group) received symptomatic treatment, normal health care and communication as usual, meanwhile they were compared with five participators (medAR group) who experienced medAR and received the same treatment as control group. We conducted a group *t* test to compare the changes in knowledge scores between our two groups following an analysis of variance test. We observed a significant effect (*P* < .001): the medAR group attained higher scores than the control group (80% and 58%, respectively; standard deviations, 4.65% and 2.81%, respectively). The results of the mean score differences using the Wilcoxon rank‐sum test were similar to those of the *t* test. Compliance behaviour of the medAR group was better than that of the control group (*P <* .05). Our findings indicate that AR can improve the shortcomings with traditional communication methods; AR can increase the effectiveness and efficiency of health‐care education and decision making based on patient family.^6^

## CONFLICT OF INTEREST

There are no conflicts of interest.

## DATA AVAILABILITY STATEMENT

The data used to support the findings of this study are available from the corresponding author upon request.
